# A revision of the diagnosis and affinities of the metriorhynchoids (Crocodylomorpha, Thalattosuchia) from the Rosso Ammonitico Veronese Formation (Jurassic of Italy) using specimen-level analyses

**DOI:** 10.7717/peerj.7364

**Published:** 2019-07-22

**Authors:** Andrea Cau

**Affiliations:** Geological and Palaeontological Museum “G. Capellini”, Bologna, Italy

**Keywords:** Bayesian phylogenetics, Middle jurassic, Metriorhynchidae, *Neptunidraco*, Italy, Specimen-level phylogenetics, Thalattosuchia

## Abstract

*Neptunidraco ammoniticus* is a thalattosuchian crocodylomorph from the Rosso Ammonitico Veronese Formation (RAVF, Middle Jurassic) of northern Italy. Erected from one partial specimen, *Neptunidraco* is pivotal in reconstructing thalattosuchian evolution, being it the oldest known member of Metriorhynchidae. Two additional RAVF thalattosuchians have been referred to *Neptunidraco*. A revised diagnosis of *N. ammoniticus* is provided here. Using a well-sampled phylogenetic data set of Crocodylomorpha, the affinities of all three RAVF thalattosuchian specimens are investigated simultaneously for the first time using parsimony tree-search strategies and Bayesian inference using the Fossilized Birth-Death with Sampled Ancestor (FBDSA) model. The results of the alternative analyses are not consistent in the placement of the RAVF specimens. The holotype of *N. ammoniticus* is consequently referred to Metriorhynchidae *incertae sedis.* The first referred specimen is recovered in various alternative placements among Metriorhynchoidea. The third and most fragmentary specimen is recovered as a crocodylomorph of uncertain affinities in the parsimony analysis and in the undated Bayesian analysis, and a metriorhynchoid sister taxon of the second RAVF specimen in the tip-dated Bayesian analysis. Only a subset of the results in the parsimony-based analyses supports the referral of the latter two specimens to *Neptunidraco*. The unusually high rate of morphological divergence for the *Neptunidraco* branch, inferred in previous iterations of the Bayesian inference analyses but not recovered in the novel analysis, was likely an artifact of the *a priori* constraint of all RAVF thalattosuchians into a single taxonomic unit, and of the arbitrarily fixed tip-age priors for the terminal taxa. These results confirm the utility of specimen-level morphological analysis and of combined tree-search strategies for inferring the affinities and the inclusiveness of fragmentary but significant fossil taxa, and reinforce the importance of incorporating stratigraphic uncertainty as prior in tip-dated Bayesian inference analyses.

## Introduction

**Figure 1:**
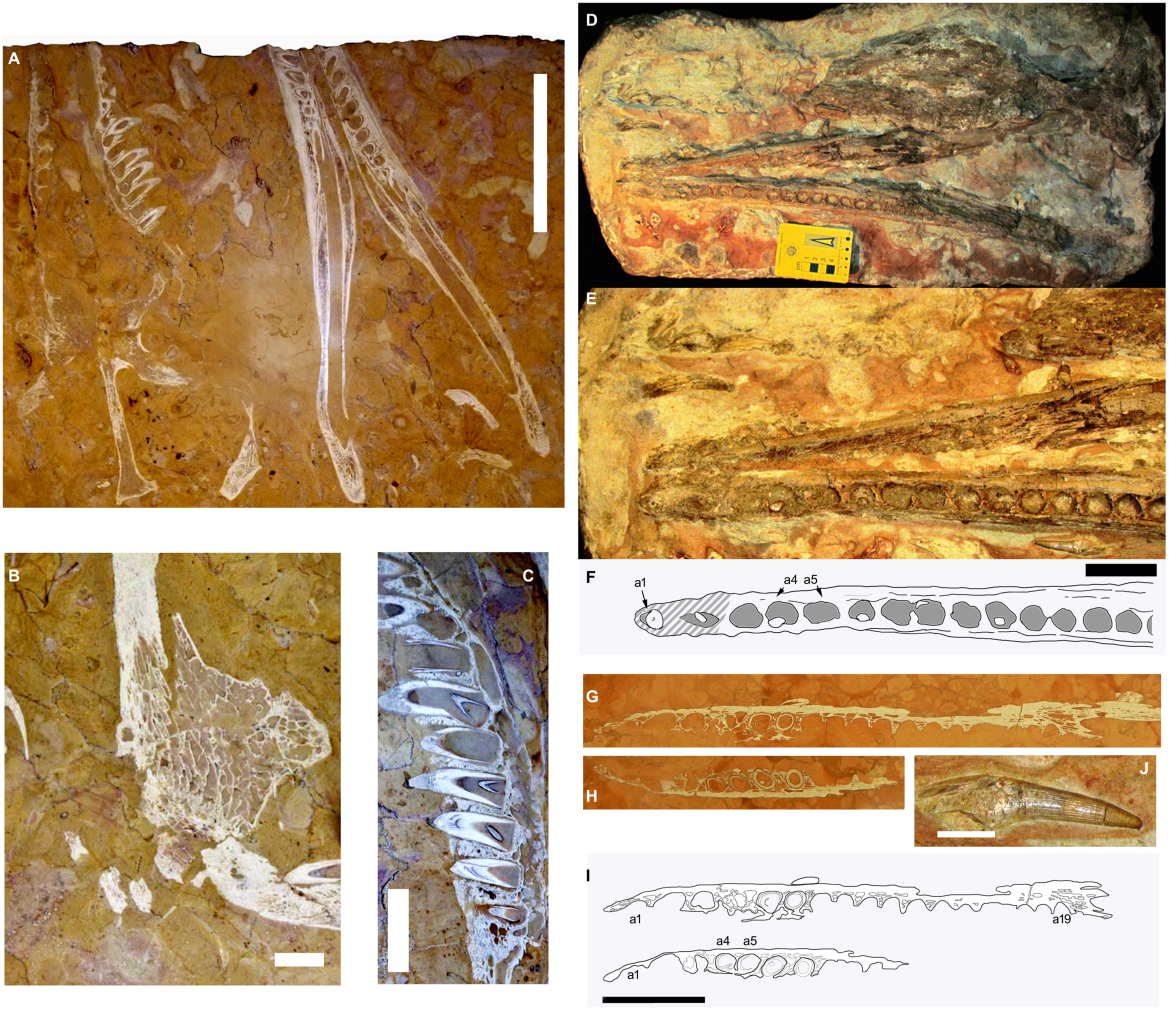
The RAVF thalattosuchian fossil record. (A–C) *Neptunidraco ammoniticus* type specimen. (A, B) MGGC 8846/1UCC123b, skull and mandible (A) and detail of prefrontal (B). (C) MPPPL 35. Detail of exposed maxillary dentition. (D–F, J) MPG 6552. (D) whole specimen, (E, F) close up of mandible, (J) isolated tooth. (G–I) FMCR SP3839. (G, H) close ups of exposed slices of same dentary ramus. (I) same as (G, H), with (H) vertically reversed to help comparison. Scale bars: 20 cm (A), 4 cm (B–I), 2 cm (J). Image copyright, Andrea Cau.

The thalattosuchian fossil record from the Rosso Ammonitico Veronese Formation (RAVF, Middle-Upper Jurassic) of the Veneto Region (northern Italy) is represented by a very limited number of specimens collected between the 18th and the 20th Centuries (Cau, 2014; Cau & Fanti, 2011; Cau & Fanti, 2015). The only valid taxon is *Neptunidraco ammoniticus* Cau & Fanti (2011), based on a specimen which includes a partial skull and mandible (both missing the rostral end) semi-articulated to the cervical series, and is dated to the latest Bajocian-earliest Bathonian (Cau & Fanti, 2011). This specimen (Figs. 1A–1C) is preserved as a series of slab slices, exposing various sections of the skeleton, a preservation which prevents a proper interpretation of the three-dimensional morphology of many elements (Cau & Fanti, 2011). A second specimen, MGP 6552, formerly referred to “*Steneosaurus barettoni*”, is an articulated skull and mandible which suffered significant dorsoventral compression and with some elements represented only as natural impressions on the slab (Figs. 1D, 1E and 1J). This specimen cannot be precisely dated along the Rosso Ammonitico Veronese Formation succession (see historical and taxonomic revision in Cau, 2014). Based on parsimony phylogenetic analysis, Cau (2014) referred this second specimen to *Neptunidraco sp.* (note that the specimen was erroneously labelled as “*Neptunidraco ammoniticus*” in Cau & Fanti, 2015, fig. 1: but see taxonomic discussion in Cau, 2014). An additional—very fragmentary—specimen is represented by a series of slab slices of a crocodylomorph mandible from the Rosso Ammonitico Veronese Formation (presumably, the lower unit of the Formation, Bathonian) (Bizzarrini, 1995). The specimen, housed in the Museo Civico of Rovereto (Trento Province, northern Italy) under accession number FMCR SP3839 (Figs. 1G and 1H), shares metriorhynchid and geosaurine synapomorphies and is indistinguishable in the overlapping elements from the other two Rosso Ammonitico Veronese Formation specimens (Cau, 2014): although its referral to *Neptunidraco* was based on both stratigraphic and morphological arguments, the affinities of this specimen have never been tested using a quantitative analysis.

Although fragmentary, *Neptunidraco* is a valid taxon, based on the unique combination of craniomandibular features, distinguishing it from all other members of Metriorhynchoidea (Cau & Fanti, 2011). The phylogenetic position of this taxon among thalattosuchians was investigated using both parsimony and model-based (Bayesian inference) methods (Cau, 2014; Cau & Fanti, 2011; Cau & Fanti, 2015), which consistently confirmed its placement among basal (non-Geosaurini) geosaurine metriorhynchids (see also, Young et al., 2014; Ösi et al., 2018). This taxon is particularly significant among metriorhynchids because its age precedes those of all other geosaurines and metriorhynchids (Cau & Fanti, 2011) and, at the same time, it shows a mix of metriorhynchid and geosaurine symplesiomorphies combined with features otherwise seen in later and more derived geosaurin taxa (Cau, 2014). Although all phylogenetic analyses published support the basal geosaurine placement for the Italian taxon as the best interpretation of the available evidence, the mosaic of features in *Neptunidraco* may support alternative suboptimal placements (e.g., a position closer to Geosaurini; see discussion in Cau, 2014). It is noteworthy that all the competing scenarios require a comparable level of homoplasy in the craniomandibular features that define the main geosaurine lineages (Young & Andrade, 2009; Young et al., 2010; Young, Bell & Brusatte, 2011; Young et al., 2012; Cau, 2014; Foffa et al., 2017). Based on the results of a Bayesian inference analysis, Cau & Fanti (2015) inferred for the lineage leading to *Neptunidraco* an unusually high rate of morphological divergence compared to other thalattosuchian branches.

Although not tested by Cau & Fanti (2015), the significantly high divergence rate inferred for *Neptunidraco* in their tip-dated Bayesian analysis might have been biased by the use of a composite taxonomic unit scored from both the holotype of *N. ammoniticus* and MGP 6552, instead of scoring the two specimens as separate (and potentially diachronous) units: although often assumed only implicitly, *a priori* definitions on the inclusiveness and composition of a taxon may have a significant yet unpredictable impact on the the result of the quantitative analyses incorporating that taxonomic unit. As discussed by Tschopp & Upchurch (2018), specimen-level phylogenetic analysis represents the most rigorous approach for testing hypotheses on the alpha taxonomy of fossil taxa. Although the discussion of Tschopp & Upchurch (2018) focused on parsimony analysis only, Cau (2017) implemented that approach using tip-dated Bayesian inference methods which simultaneously integrates morphological diversity and stratigraphic distribution. Here, the phylogentic affinities of the three Rosso Ammonitico Veronese Formation metriorhynchoids are investigated for the first time including all specimens simultaneously in a quantitative analysis which also takes into account the uncertainty in their stratigraphic positions, and comparing the results of the analyses performed using different tree search strategies (see Madzia & Cau, 2017; Smith, 2019, for a discussion on the utility of comparing alternative tree search strategies).

## Material and Methods

The phylogenetic affinities of the RAVF thalattosuchians were tested using one of the most recently updated data sets for Crocodylomorpha (Ösi et al., 2018, supplementary material: data set 1). Character statements and settings follow Ösi et al. (2018). The data matrix was analyzed using unweighted parsimony, Implied Weighted parsimony (Goloboff, 1993; Goloboff, 1995) and model-based Bayesian inference methods: the results of the analyses using these alternative methods were compared (see discussion in Madzia & Cau, 2017). The taxon “*Neptunidraco ammoniticus*” included in the original data set of Ösi et al. (2018) was removed and replaced by three taxonomic units, that were scored, respectively, on the holotype of *N. ammoniticus* (Cau & Fanti, 2011; material housed in the MGGC and MPPPL collections), on specimen MGP 6552 (Cau, 2014), and on FMCR SP3839 (Bizzarrini, 1995; Cau, 2014). The latter specimen is included in a numerical analysis for the first time. The holotype of *N. ammoniticus* was positively scored for 22 character statements (5% of total character list), MGP 6552 for 45 (10% of total character list), and FMCR SP3839 for 15 character statements (3% of total character list). The character scores of the three specimens do not completely overlap, and there are no differentiating scores (i.e., characters with states scored differently in at least two of the RAVF specimens, see below). Note that the taxonomic unit “*Neptunidraco ammoniticus*” in Ösi et al. (2018) is stated to be scored solely on information from Cau & Fanti (2011) and does not include information from either MGP 6552 or FMCR SP3839 (Ösi et al., 2018, supplementary information).

The character scoring for the three taxonomic units added in the data set followed a conservative approach, and some character states that were scored for *Neptunidraco* in previous analyses (e.g., Cau & Fanti, 2011; Cau, 2014; Ösi et al., 2018) have been re-scored here as “unknown” since it cannot unambiguously be excluded that the preserved conditions represent taphonomic or preparation artifacts. For example, although the inflection point of the prefrontal (*sensu* Wilkinson, Young & Benton, 2008) in the holotype of *N. ammoniticus* appears inclined relative to the anteroposterior axis of the skull (Fig. 1B), and was scored accordingly in the phylogenetic analyses used in Cau & Fanti (2011), and in Cau (2014), the majority of the skull elements are partially disarticulated, thus preventing the accurate determination of their original orientations. Accordingly, the character statement describing this feature in *N. ammoniticus* has been re-scored as unknown.

The taxonomic unit “*Machimosaurus rex*” (Fanti et al., 2016) was also re-scored based on information obtained from first-hand examination of the type specimen. The updated character score of *M. rex* and of the three Italian metriorhynchids in the data set of Ösi et al. (2018) is provided in the Supplemental Information.

The parsimony phylogenetic analysis was performed in TNT vers.1.5 (Goloboff, Farris & Nixon, 2008). Maxtree was set at = 50000. The heuristic search analysis performed 1,000 tree-bisection-reconnection analyses, saving all shortest trees found. All characters were set as having equal weight, and ordered characters followed the settings in Ösi et al. (2018). In order to test whether the relationships among thalattosuchians are biased by assumptions on character weighting settings, four replications of the analysis were performed using the “Extended Implied Weighting” function in TNT vers. 1.5, setting, alternatively, the concavity parameter *K* at values, 3, 6, 9, and 12 (Goloboff, 1993; Goloboff, 1995). The results of different runs were compared in order to spot, and prioritize, the groupings that are consistently being reconstructed, and which thus are less biased by *a priori* assumptions on character homoplasy weighting (see discussion in Madzia & Cau, 2017).

Bayesian inference analysis integrating morphological and stratigraphic information was performed in BEAST vers. 2.4.4 (Drummond et al., 2012; Bouckaert et al., 2014), implemented with the packages for the analysis of morphological characters, using the model of Lewis (2001), and for sampling potential ancestors among the ingroup (Gavryushkina et al., 2014). Bayesian analysis used the same data matrix used in the parsimony analysis. Stratigraphic information was converted to geochronological ages (million years before the present) and used as tip age prior. Age uncertainty (due to absence of direct absolute dating of the specimens, or to uncertainty in the absolute age of the fossil-bearing levels including the OTUs) was incorporated in the tip age prior setting, using for each OTU a uniform prior spanning the shortest age range that unambiguously constraints that OTU. Stratigraphic ranges for taxa are based on Ösi et al. (2018) supplementary material. The stratigraphic range of the holotype of *N. ammoniticus* was set as the uniform prior spanning the lower unit of the Rosso Ammonitico Veronese Formation (Bajocian-Bathonian, (Cau & Fanti, 2011). Since the precise stratigraphic positions of MGP 6552 and FMCR SP3839 along the Rosso Ammonitico Veronese Formation are unknown (Cau, 2014), the age priors for the latter two taxonomic units were conservatively set as the uniform prior spanning the whole extent of the Formation (Bajocian-Tithonian; (Cau & Fanti, 2011). The extant taxa included in the data set** were used to calibrate the height for the tip-date setting in the Bayesian analysis: the uniform prior setting used for incorporating uncertainty in the age of the fossil taxa requires at least one terminal taxon to have the tip age fixed to a value (see Gavryushkina et al., 2014; Gavryushkina et al., 2017; and Cau, 2017). Numerical ages of stages are based on the International Chronostratigraphic Chart (Cohen et al., 2018). Bayesian inference analysis was performed following the method discussed by Lee et al. (2014a), using implementations discussed by Lee et al. (2014b) and the tree model sampling ancestors introduced by Gavryushkina et al. (2014) and Gavryushkina et al. (2017). The Lewis (2001) model was conditioned to variable characters only (implementation included in BEAST 2.4.4) because the character list of Ösi et al. (2018) does not sample among autapomorphies of the included species. In the analysis, rate variation across traits was modeled using the multi-gamma parameter (default model and unique implemented for the analysis of morphological data in BEAST). The rate variation across branches was modeled using the relaxed log-normal clock model, with the number of discrete rate categories that approximate the rate distribution set as *n*-1 (with *n* the number of branches), the mean clock rate using default setting, and not setting to normalize the average rate. The Sampled Ancestor Fossilized Birth Death Skyline Model (Gavryushkina et al., 2014) was used as tree model, as it does not require a uniform sampling rate in tree estimation and encompasses the heterogeneous stratigraphic distribution of the terminal tips that usually characterizes the fossil record (see Madzia & Cau, 2017). The analysis performed a single run of 40 million generations, with burnin set at 20%. A previous analysis using an analogous setting also tested an alternative run involving four replicates of 10 million generations each, and did not find significant differences between the results of the two runs (Madzia & Cau, 2017). The Maximum Clade Credibility Tree (MCCT, the tree with the highest sum of posterior probability values at branches) is used to represent the inferred relationships, with posterior probability of branches measured according to their frequency in the post-burnin sample of reconstructed trees.

The Bayesian inference analysis performed here differs from the analogous analysis in Cau & Fanti (2015) for three significant elements:

1. *Neptunidraco* is here split into three taxonomic units (the holotype of *N. ammoniticus*, and the two referred specimens) and its monophyly is not assumed *a priori* (as instead assumed in Cau & Fanti, 2015), who based it on the result of the parsimony analysis of Cau (2014). This procedure allows to test all possible relationships for the Rosso Ammonitico Veronese Formation metriorhynchoids (i.e., if they form a clade excluding all other included taxonomic units, vs if they form an anagenetic series, or, instead, if they do not form a clade excluding other members of Metriorhynchidae).
2. The age uncertainty of each taxon is incorporated in the analysis: age prior of all fossil taxa is defined as a uniform range including the absolute age limits of the shortest stratigraphic range unambiguously including any taxon (see Cau, 2017). This approach differs from that followed in Cau & Fanti (2015), who used for each tip a fixed age prior defined arbitrarily by the mean value of the stratigraphic uncertainty of each taxon.
3. The tree model used here discriminates between anagenetic and cladogenetic patterns of evolution, and thus may test whether some of the taxonomic units included (in particular, the specimen-level units like the three specimens of *Neptunidraco*) eventually form anagenetic sequences (see discussion in Cau, 2017) for the implications on both branch duration and taxonomic diversity inference in a specimen-level analysis of fossil). The analysis in Cau & Fanti (2015) followed the method of Lee et al. (2014a) and Lee et al. (2014b) and a previous version of BEAST which did not implement the Sampled Ancestor Fossilized Birth Death Skyline Model (Gavryushkina et al., 2014), and thus was *a priori* constrained to reconstruct exclusively cladogenetic frameworks.

In order to test whether the relationships inferred in the Bayesian inference analysis are significantly biased by the stratigraphic data, a second Bayesian analysis was performed, using the same settings as the first analysis but excluding the stratigraphic information.

Thalattosuchian beta taxonomy follows Young & Andrade (2009), Young et al. (2010), Cau & Fanti (2011), and Young et al. (2012).

The terms “geosaurine” refers to a member of Geosaurinae, whereas “geosaurin” refers to a member of Geosaurini.

Abbreviations: FMCR SP, Fondazione Museo Civico di Rovereto, Sala di Paleontologia, Rovereto, Italy; MGGC, Museo Geologico “Giovanni Capellini”, Bologna, Italy; MGP, Museo Paleontologico Universitario di Padova, Padova, Italy; MPPPL, Museo Paleontologico e della Preistoria “Leonardi”, Ferrara, Italy.

## Results

### Systematic paleontology

**Table:** 

Crocodylomorpha Hay, 1930
Thalattosuchia Fraas, 1901
Metriorhynchoidea (Fitzinger, 1843)
Metriorhynchoidea *incertae sedis*

**Material.** FMCR SP3839; MGP 6552.****

**Remarks.** Although the two specimens from the RAVF show some of the mandibular features listed in the differential diagnosis of *N. ammoniticus* (see below), these are widespread among Metriorhynchoidea, and are not sufficient for unambiguously referring FMCR SP3839 and MGP 6552 to that species. The conflicting results of the alternative phylogenetic analyses (see below) demonstrate the unstable placement of these fragmentary specimens, which are conservatively referred solely to Metriorhynchoidea. Although the parsimony analyses recovered also some non-thalattosuchian placements for FMCR SP3839 among the shortest trees found, the referral of the latter specimen to Metriorhynchoidea is considered the most plausible interpretation of its morphology and stratigraphic setting (see discussion, below).

Metriorhynchidae Fitzinger, 1843**

*Neptunidraco* Cau & Fanti, 2011

**Type species.** *Neptunidraco ammoniticus* Cau & Fanti, 2011.

*Neptunidraco ammoniticus* Cau & Fanti, 2011

**Type material.** MGGC 8846/1UCC123b, MGGC 8846/1UCC123a, MPPPL 35, MPPPL 39 (Cau & Fanti, 2011). A single, partially-preserved and semi-articulated individual.

**Figure 2:**
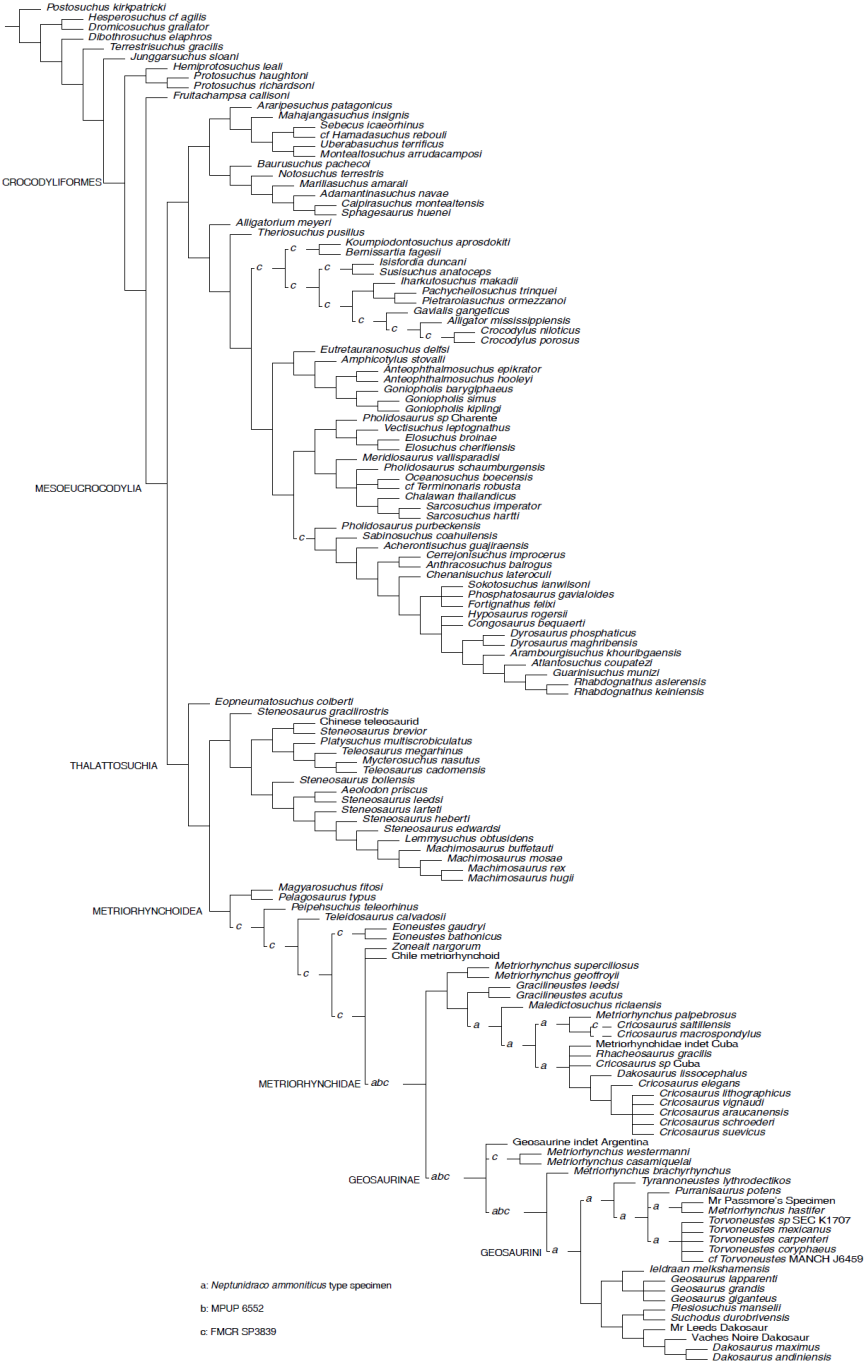
Parsimony Analysis. Reduced Strict Consensus of the shortest trees found by the equal weight parsimony analysis, after a posteriori pruning of the RAVF specimens: the alternative positions recovered for the Italian specimens are indicated by the letters (a–c) above the branches.

**Diagnosis of *N. ammoniticus* (emended from Cau & Fanti, 2011)**. Metriorhynchoid crocodylomorph differing from all other metriorhynchoids by the following combination of features: (1) prefrontal drop-shaped in dorsal view (Fig. 1B); (2) postorbital ramus of frontal forms a 60–70° angle with the anteroposterior axis of the fronto-parietal supratemporal bar; (3) in dorsal view, postorbital distinctly bends caudally at about 110–120° at the level of the rostrolateral margin of the supratemporal arch; (4) more than eleven alveoli in both maxilla and dentary, with at least ten adjacent to the mandibular symphysis, and the posteriormost eight alveoli adjacent to the splenial; (5) maxillary and dentary alveoli which are separated by uniformly narrow interalveolar spaces being about 1/3 of the mesiodistal diameter of adjacent alveoli.

**Remarks.** The original diagnosis of *N. ammoniticus* included additional features which are here excluded from the diagnosis, being likely due to a combination of taphonomic and preparation artifacts in the partially disarticulated holotype skull (i.e., character “1.”, characters “3.” and “5.”in Cau & Fanti, 2011: 559). The combination of features listed in the emended diagnosis differentiates *Neptunidraco* from all other named “genus-level” metriorhynchoid taxa (Andrews, 1913; Andrade et al., 2010; Young et al., 2010; Young et al., 2012; Young et al., 2014; Foffa et al., 2017; see Cau & Fanti, 2011).

List of character statements scored for the Rosso Ammonitico Veronese Formation thalattosuchians in the character list of Ösi et al. (2018)

Character statement 8: Rostrum, in dorsal view –amblygnathy (“bullet-shaped”, with the rostrum retaining its width along almost all its length): absent.

The “amblygnathous” condition was described and illustrated by Young et al. (2012), who found it as a derived morphology of the snout in *Dakosaurus*. Although preserved uniquely as an impression of the dentigerous margin on the slab, the snout of MGP 6552 is proportionally more elongate than in *Dakosaurus*, and does not fit the relatively wider and “bullet-shape” morphology of the latter genus. This character cannot be scored for either *N. ammoniticus* type or FMCR SP3839.

Character statement 9: Rostrum, presence of distinct flattening of the cranial rostrum dorsal surface and symphyseal dentary ventral surface: absent.

The symphyesal end of the dentary is exposed in both hemimandibles of MGP 6552, respectively in dorsal view (left ramus) and medial view (right ramus). The derived condition of the character describes an almost planar dentary symphyseal region, a condition which can unambiguously be negated for the Italian specimen. This character cannot be scored for either *N. ammoniticus* type or FMCR SP3839.

Character statement 52: Nasals, posterior portion at the midline: has a concavity at the midline, or a ’midline trench’.

Although the whole specimen is dorsoventrally compressed, the posterior end of the nasals in MGP 6552 shows a distinct midline depression running anteroposteriorly and bordered laterally by the moderately convex dorsal surface of each nasal. It is unlikely that this condition is a preservational artifact (Cau, 2014). This character cannot be scored for either *N. ammoniticus* type or FMCR SP3839.

Character statement 54: Nasal-prefrontal contact: present.

The prefrontal-nasal contact is preserved in the type specimen of *N. ammoniticus* (MGGC 8846/1UCC123b) and in MGP 6552. This character cannot be scored for FMCR SP3839.

Character statement 85: Supratemporal fenestrae, presence: present as an evident fenestra.

Although incompletely preserved, the supratemporal fenestrae are preserved in the holotype of *N. ammoniticus* (better visible in MGGC 8846/1UCC123a, in MPPPL 35, and in MPPPL 39) and in MGP 6552. This character cannot be scored for FMCR SP3839.

Character statement 89: Supratemporal fossa/fenestra, anterior margin shape, anterolateral expansion: absent.

The derived condition of this character statement is positively scored when the anterior margin of the supratemporal fossae are noticeably inclined anterolaterally, such that the anterolateral corners of the supratemporal fossae are more anterior than the anteromedial corners of the supratemporal fossae. This condition is absent in the holotype of *N. ammoniticus*: the exposed margins of the supratemporal fossa consistently curve posterolaterally relative to the anteromedial corner of the fossa (i.e., MPPPL 35, and MPPPL 39). This character cannot be scored for MGP 6552 neither FMCR SP3839.

Character statement 90: Supratemporal fenestra, overall anteroposterior elongation: length is either less than, or approximately sub-equal to the anterior width.

&**

*Character statement 91: Supratemporal fenestra, overall anteroposterior elongation: length is either less than, or approximately sub-equal to the width at the middle of the fenestra* (±*25*%).

Although the supratemporal fenestrae are only partially preserved in the holotype of *N. ammoniticus*, and suffered some taphonomic deformation due to dorsoventral compression, based on the almost complete preservation and articulation of the postorbital margin of the fenestra (visible in MGGC 8846/1UCC123a), it is highly unlikely that the original length of the fenestra did exceed 110% of its width. This character cannot be scored for MGP 6552 or FMCR SP3839.

Character statement 95: Supratemporal arch, medial margin in dorsal view: not convex.

In the slabs of the holotype of *N. ammoniticus* where it is exposed, the medial margin of the supratemporal arch describes a continuous concavity (i.e., MGGC 8846/1UCC123b and MGGC 8846/1UCC123a). This character cannot be scored for MGP 6552 or FMCR SP3839.

*Character statement 98: Prefrontal, dorsal surface lateral development: enlarged (extending laterally over the orbit by* >*15% of its width).*

Although the lateral extent of the prefrontal relative to the orbit cannot be determined in the holotype of *N. ammoniticus*, the preserved bone is clearly expanded laterally beyond the lateral margin of the maxillary tooth row and the posterolateral margin of the nasal (MGGC 8846/1UCC123b, MGGC 8846/1UCC123a, MGGC 8846/1UCC123a, MPPPL 35, MPPPL 39), thus recalling the condition in metriorhynchids more than those in other crocodylomorphs (e.g., Andrews, 1913; Young et al., 2010). This character is thus scored as having the most derived condition in the holotype of *N. ammoniticus*. Although the lateral margin of the prefrontal is eroded in MGP 6552, the preserved part of the bones is expanded laterally and clearly differs from the plesiomorphic state of this character: the specimen is thus scored as “1/2”. This character cannot be scored for FMCR SP3839.

Character statement 100: Prefrontal, shape in dorsal view: teardrop-shaped.

The prefrontal is partially exposed in the holotype of *N. ammoniticus* and is best visible in MGGC 8846/1UCC123b. The prefrontal in MGP 6552 is partially preserved, with the lateral margin partially eroded and preserved as a shallow impression on the slab (Cau, 2014). The preserved part of the prefrontal in the latter and the slab impression in the former closely recall the “teardrop” shape of most metriorhynchid prefrontals, and differ from the more irregular outline of other crocodylomorphs (e.g., Andrews, 1913; Young et al., 2010; Young et al., 2013). In conclusion, taking into account the different preservations, both specimens can be positively scored for the presence of a teardrop-shaped prefrontal. This character cannot be scored for FMCR SP3839.

Character statement 111: Frontal, angle between posteromedial and posterolateral processes: approximately 70–60°.

Although partially preserved, both posterolateral processes of the frontal in the holotype of *N. ammoniticus* are symmetrically oriented relative to the posteromedial process, and form with the latter an angle of about 60–65° (MGGC 8846/1UCC123a, MPPPL 35). This indicates that the original orientation of these elements is preserved in the specimen. This character cannot be scored for MGP 6552 or FMCR SP3839.

Character statement 117: Postorbital, shape in dorsal view: the outer margin is convex where the postorbital curves posteriorly forming the supratemporal arch.

The dorsal margin of the right postorbital ramus is preserved in the holotype of *N. ammoniticus* and bears an abrupt posterior bent at the level of the anterolateral corner of the supratemporal fenestra (MGGC 8846/1UCC123b). This character cannot be scored for MGP 6552 neither FMCR SP3839.

Character statement 120: Supratemporal arch (= upper temporal bar), relative participation of the postorbital: extensive, postorbital represents approximately 50% (or more) of the bar.

The extent of the supratemporal arch is almost completely preserved in the holotype of *N. ammoniticus*. In particular, the frontal-postorbital suture and the postorbital-squamosal articulations are visible in MGGC 8846/1UCC123b and in MPPPL 39, confirming that the postorbital forms more than half of the extent of the supratemporal arch. This character cannot be scored for MGP 6552 neither FMCR SP3839.

Character statement 231: Mandibular rami, presence of a sharp dorsal inclination: absent.

The right hemimandible of MGP 6552 is partially preserved and exposed in medial view, although overlapped by the skull in its posterior third (Cau, 2014). Nevertheless, the exposed part of the mandible shows that immediately posterior to the mandibular symphysis the mandible gently rises dorsally such that the ventral margin of the dentary (along with angular) is uniformly deflected dorsally (i.e., lacking a distinct kink along the ventral margin). This character cannot be scored for the holotype of *N. ammoniticus* or FMCR SP3839.

Character statement 232: Mandible, orientation of hemimandibles at their medial contact: evidently acute angle, hemimandibles meet at approximately 45° of each other, or less.

Although the rostral end of the mandible in the holotype of *N. ammoniticus* is lost, the preserved parts of the two hemimandibular rami are preserved in close contact and suffered limited dislocation from each other, providing an accurate depiction of the original relative orientation of the two rami in life (MGGC 8846/1UCC123a, MPPPL 35), and showing that the two hemimandibles met medially at a narrowly acute angle. A comparable condition is preserved in MGP 6552 (Cau, 2014). This character cannot be scored for FMCR SP3839.

Character statement 237: Anterior mandible (dentary), dorsal margin of the anterior portion compared to the dorsal margin of the posterior portion: horizontal (in the same plane).

& **

Character statement 246: Dentary, ventral margin strongly curved: no.

The right hemimandible of MGP 6552 is partially preserved and exposed in medial view, although overlapped by the skull in its posterior third (Cau, 2014). Nevertheless, the exposed part of the mandible shows that the dorsal and ventral margins of the dentary are uniformly straight along their whole extent. These characters cannot be scored for the holotype of *N. ammoniticus* or FMCR SP3839.

Character statement 238: Anterior mandible (dentary), in dorsal or ventral view: outer margin converging towards tip or parallel.

The left hemimandible of MGP 6552 is preserved and exposed in dorsal view (Cau, 2014). Although the occlusal surface of the rostralmost end (at the level of the first two alveoli) is eroded, the lateral surface of the bone is better preserved and shows that the outer margin was subparallel to the medial margin, and converged toward the tip in the rostralmost termination. The same condition is visible in the preserved dentary of FMCR SP3839. This condition cannot be scored for the holotype of *N. ammoniticus*.

Character statement 240: Mandibular symphysis, length: symphysis between 30 and 45% of mandible length.

The left hemimandible of MGP 6552 is preserved and exposed in dorsal view, with only the retroarticular process missing (Cau, 2014). The dentary symphysis extends for about 36–40% of the preserved length of the hemimandible. It is thus plausible to assume that the symphysis extended for less than 40% of the original length of the mandible. Even taking into account the missing distal part of the mandible and its contribution to overall mandible length in metriorhynchoids (e.g., Andrews, 1913; Young et al., 2012), it is unlikely that the symphysis extended for less than 30% of the whole mandible length. This condition cannot be scored for the holotype of *N. ammoniticus* or FMCR SP3839.

Character statement 241: Mandibular symphysis, depth: narrow (4.5–6% of mandible length).

The right hemimandible of MGP 6552 is partially preserved and exposed in medial view, although overlapped by the skull in its posterior third (Cau, 2014). Nevertheless, the exposed part of the mandible shows that the average depth of the mandibular symphysis is around 5% of the inferred length of the mandible (see character 240, above). This character cannot be scored for the holotype of *N. ammoniticus* or FMCR SP3839.

*Remarks.* Both scores for characters 240 and 241 should be considered as tentative estimations, given the bad preservation of the posterior end of the mandible, the absence of the retroarticular process and the overall compression of the specimen, which prevent an accurate determination of the mandibular length. To test whether these two character state estimations significantly bias the phylogenetic affinities of MGGP 6552, an alternative parsimony analysis was performed, setting conservatively both character states as “unknown”. The results of this test were compared with those of the analysis setting positively both characters.

Character statement 252: Splenial, involvement in mandibular symphysis: extensive (greater than, or equal to, 15% of symphysis length).

The right hemimandible of MGP 6552 is partially preserved and exposed in medial view, although overlapped by the skull in its posterior third (Cau, 2014). Nevertheless, the exposed part of the mandible shows that the splenial extends rostrally overlapping the posteriormost eight alveoli, contributing for about one fifth of the mandibular symphysis. Although this character cannot be scored for the holotype of *N. ammoniticus* (missing the rostral part of the mandible) or FMCR SP3839, in the former the splenial extends rostrally to the level of the eight posteriormost alveoli (MGGC 8846/1UCC123a, MPPPL 35), as in MGP 6552, suggesting that the splenial contributed to a comparable extent of the symphysis.

Character statement 270: Premaxilla, alveolar count: three or fewer alveoli.

The natural casts of the alveoli on the slab of MGP 6552 shows that each premaxilla bears three teeth (Cau, 2014). This character cannot be scored for the holotype of *N. ammoniticus* or FMCR SP3839.

Character statement 278: Dentary, alveolar count: 19–15 alveoli.

Both MGP 6552 and FMCR SP3839 bear 19 alveoli in the dentary. This character cannot be scored for the holotype of *N. ammoniticus.*

Character statement 280: Maxillary interalveolar spaces, relative size: interalveolar spaces are variable in size, some are similar in length to the adjacent alveoli, while others are approximately half the length of the immediately adjacent alveoli (especially towards the end of the maxillary tooth row)

And**

Character statement 283: Dentary interalveolar spaces, relative size: interalveolar spaces are variable in size, some are similar in length to the adjacent alveoli, while others are approximately half the length of the immediately adjacent alveoli.

The interalveolar spaces in both maxilla and dentary of the holotype of *N. ammoniticus* (visible in MGGC 8846/1UCC123a and MPPPL 35) are preserved as longitudinal sections of the tooth-bearing bones. This condition prevents a direct determination of the above-listed states in this taxon, but allows to exclude that the alveoli were widely spaced by interalveolar spaces larger than the adjacent tooth sockets. This feature is consistently present in all tooth-bearing bones exposed, and cannot be an artifact of the peculiar preservation of the specimen. The interalveolar spaces in *Neptunidraco* and MGP 6552 appear intermediate in size between the narrower condition present in some geosaurins (e.g., *Dakosaurus*, Young et al., 2012) and the plesiomorphic condition shared by the majority of non-geosaurin thalattosuchians, a condition comparable to some geosaurines like *Tyrannoneustes* (Young et al., 2014; Foffa & Young, 2014), which is scored as “0” for the two above mentioned character statements in the data set of Ösi et al. (2018). Accordingly, the RAVF specimens are scored as state “0” of these character statements. The character statement 280 cannot be scored in MPG 6552 or FMCR SP3839. The preservation of FMCR SP3839 is similar to the holotype of *N. ammoniticus*, and shows comparably-wide interalveolar spaces in the dentary: accordingly, it is scored as showing the plesiomorphic state for character 283.

Character statement 282: First dentary alveolus, orientation: dorsally orientated.

The base of the first dentary tooth in MGP 6552 is preserved *in situ*, and indicates that the tooth was oriented dorsally, perpendicularly to the alveolar margin. This character cannot be scored for the holotype of *N. ammoniticus* or FMCR SP3839. **

Character statement 293: Dentary alveoli, number of alveoli adjacent to the mandibular symphysis: between 10 and 14.

The rostral end of the mandible in the holotype of *N. ammoniticus* is missing, thus the exact number of alveoli adjacent to the symphysis is unknown, although, based on the preserved rami, this number was greater than nine. In MGP 6552, the left hemimandible is exposed in dorsal view, and shows 13 alveoli adjacent to the symphysis. This character cannot be scored for FMCR SP3839. **

Character statement 307: Mid to posterior mandibular teeth, transverse section: transverse section circular to subcircular, without significant lateral compression.

&**

Character statement 308: Dentition, presence of apicobasal facets on the labial sufrace: absent, either lacking facets, or facetted into 4–5 indistinct planes.

& **

Character statement 309: Dentition, presence of laminar teeth: absent.

A few mandibular teeth are preserved adjacent to the posterior half of the dentary of MGP 6552, and one posterior dentary tooth, missing the tip, is still in its alveolus. All these teeth lack evident transverse compression of the crown or apicobasal faceting of the crown surface. These characters cannot be scored for FMCR SP3839. Some teeth are still *in situ* in the maxilla and dentary of the holotype of *N. ammoniticus*: the elliptical outline of the exposed crowns differs from the apomorphic states of these characters.

Character statement 317: Carinae, presence of keel at the edge of tooth crown: present (i.e., carinated sensu stricto, created by a smooth keel [raised ridge] on the crown edges, typically on the mesial and distal margins).

A few mandibular teeth are preserved adjacent to the posterior half of the dentary of MGP 6552, and one posterior dentary tooth, missing the tip, is still in its alveolus. The best preserved tooth shows a faint carina along the distal margin of the crown (Cau, 2014). This character cannot be scored for the holotype of *N. ammoniticus* or FMCR SP3839. **

Character statement 328: Morphology of enamel surface ornamentation, apicobasal ridges: enamel ornamentation composed of well-defined apicobasally aligned ridges that are conspicuous and are elongate; being continuous, or having long discontinuous ridges.

The best preserved teeth of MGP 6552 show a series of closely-appressed ridges oriented apicobasally and extended for at least the basal two-thirds of the crown (Cau, 2014). This character cannot be scored for the holotype of *N. ammoniticus* or FMCR SP3839. **

Character statement 329: Morphology of apical enamel surface ornamentation, macroscopic anastomosed pattern: absent.

In all preserved teeth of MGP 6552, the apical third of the crown is unornamented or is crossed by apicobasally-aligned ridges. This character cannot be scored for the holotype of *N. ammoniticus* or FMCR SP3839.

Character statement 339: Cervical vertebrae, shape: amphicoelous or amphyplatian.

Although the preserved cervical centra in the holotype of *N. ammoniticus* are variably sliced along different planes (MGGC 8846/1UCC123a, MPPPL 35, MPPPL 39), they all show flat to moderately concave intercentral facets. This character cannot be scored for MGP 6552 or FMCR SP3839. **

Phylogenetic analyses

*Parsimony analyses.* The phylogenetic analysis using TNT and equal weighting recovered >50000 shortest trees of 1484 steps each (CI = 0.4151, RI = 0.8408). The topologies of the shortest trees are in overall agreement with the results obtained by Ösi et al. (2018), with the notable exception of the position of *Neptunidraco ammoniticus* type specimen (MGP 6552 and FMCR SP3839 were not included in the original analysis), which is found in various positions among ** metriorhynchids: as sister-group of Metriorhynchidae, as a basal metriorhynchine more derived than *Gracilineustes*, or as a geosaurine outside the clade including *Dakosaurus*, *Geosaurus* and *Plesiosuchus* (Fig. 2). The specimen MGP 6552 was recovered in three alternative positions: as sister group of Metriorhynchidae, as the basalmost geosaurine or as the sister taxon of the clade including “*Metriorhynchus*” *brachyrhynchus* and Geosaurini (Fig. 2). Note that the holotype of *N. ammoniticus* is alternatively placed in these positions in a subset of the shortest trees found. Exploration of the shortest trees found showed that the specimen FMCR SP3839 was recovered in various alternative placements among Crocodylomorpha, i.e., among Eusuchia, Tethysuchia and Metriorhynchoidea (Fig. 2). In a subset of trees, FMCR SP3839 is placed as sister taxon of the holotype of *N. ammoniticus*. It is also noteworthy that in a subset of the shortest trees found, the three RAVF specimens form a clade, placed among the basalmost branches of Geosaurinae. Nevertheless, no unambiguous synapomorphy supports a monophyletic group including all RAVF specimens with the exclusion of other taxa: all features shared by them are optimized as metriorhynchid or geosaurine symplesiomorphies.

**Figure 3:**
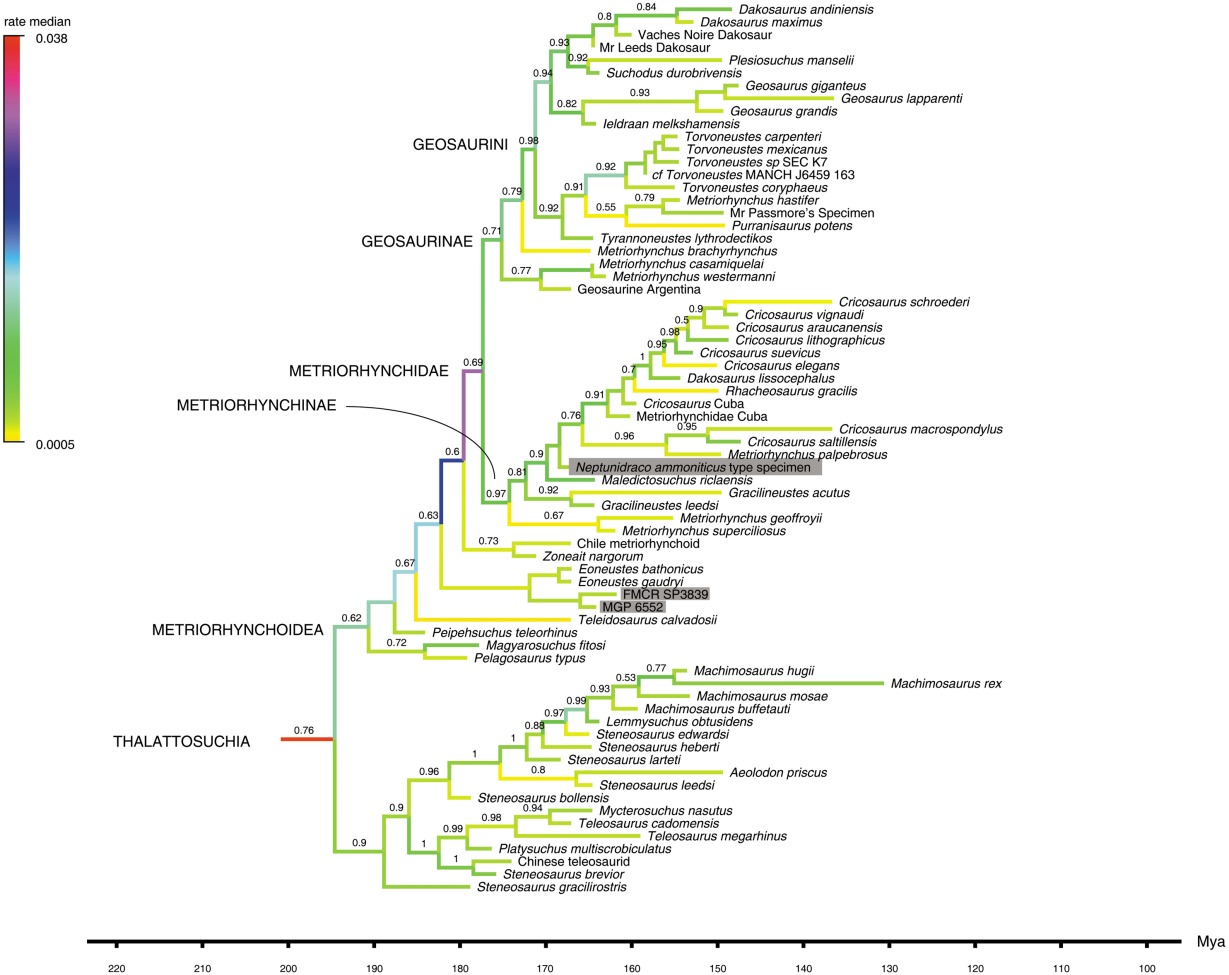
Detail of Maximum Clade Credibility Tree. Detail of the Maximum Clade Credibility Tree reconstructed by the Bayesian inference analysis, focusing on thalattosuchians. Non-thalattosuchians omitted (see complete tree in Supplemental Information). Branches colored according to median rate of morphological divergence inferred (note highest values at the root of the thalattosuchian and metriorhychid radiations). Tip ages based on median value inferred: actually recorded extent of terminal taxa not reported. Note that additional Early Cretaceous material (not included in the analysis) may extend the metriorhynchid record to the Aptian-Albian (see Chiarenza et al., 2015), and that the timing of extant clade diversification is delayed by absence in the sample of fossil members of the crown-group (see complete tree in Supplemental Information). The numbers above the branches indicate the posterior probability values >0.5. The RAVF specimens are indicated by the grey rectangles.

All the four analyses performed using the “Extended Implied Weighting” function in TNT vers. 1.5 produced the same topologies as the equally-weighted analysis.

The replication of the equal weighed parsimony analysis, setting both states for characters 240 and 241 as “unknown” in MGGP 6552, produced the same topologies recovered by the analysis using the states tentatively estimated for the two mentioned characters.

**Figure 4:**
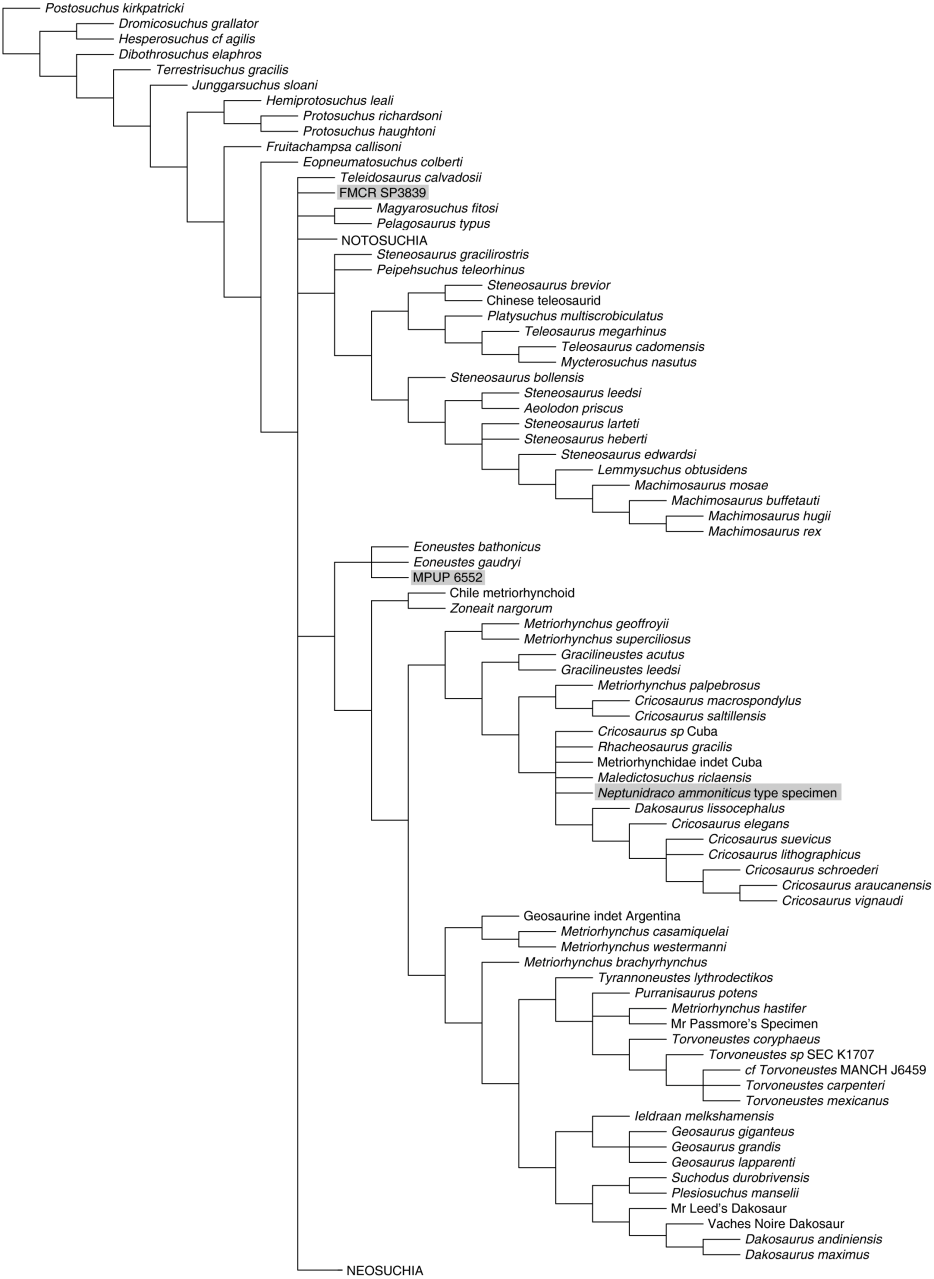
Half-compact tree of undated Bayesian inference analysis of Crocodylomorpha. Half-compact tree of the post-burnin trees sampled by the Bayesian inference analysis not integrating the stratigraphic information. RAVF specimens indicated by grey rectangles. Notosuchia and Neosuchia collapsed for brevity (see complete tree in Supplemental Information).

*Bayesian inference analysis.* The MCCT reconstructed by the tip-dated Bayesian inference analysis (Fig. 3) is in overall agreement with the strict consensus of the shortest trees found by the parsimony analysis, with the large majority of branches having a moderate to high posterior probability value. Focusing on the topology, the most notable difference from the parsimony analysis is the position of the Italian specimens, with the holotype of *N. ammoniticus* found in Metriorhynchinae (as in a subset of the shortest trees found in the parsimony analyses, Fig. 2), whereas the other two RAVF specimens are placed as sister- taxa in a lineage with the non-metriorhynchid metriorhynchoid *Eoneustes* (Young et al., 2010). The position of *N. ammoniticus* type among Metriorhynchinae is well supported (the posterior probability values, *pp*,** of the basalmost nodes of Metriorhynchinae including also to the Italian taxon range between 0.81 and 0.90), although its relationships relative to *Maledictosucus* and Rhacheosaurini are weakly supported, being based on one single feature which is also convergently developed among geosaurines (the acute angle between the posteromedial and posterolateral processes of the frontal; character statement 111.1). The sister-group relationships of the other two RAVF specimens and their affinity with *Eoneustes* are very weakly support (*pp* <0.5), and are not based on unambiguous synapomorphies (as determined by character optimisation in TNT and using the enforced MGGC topology). The result of the second Bayesian-based analysis (not integrating the stratigraphic age of the taxa in tree reconstruction) confirms both the placement of *N. ammoniticus* holotype in Metriorhynchinae and the sister taxon relationship between MGP 6552 and *Eoneustes*, but does not support the sister taxon relationship between MGP 6552 and FMCR SP3839 (Fig. 4). The half-compact consensus of the post-burnin trees inferred by the second Bayesian inference analysis places FMCR SP3839 in a large unresolved polytomy among mesoeucrocodylians, a result similar to the strict consensus tree of the topologies inferred by the parsimony analyses.

**Figure 5:**
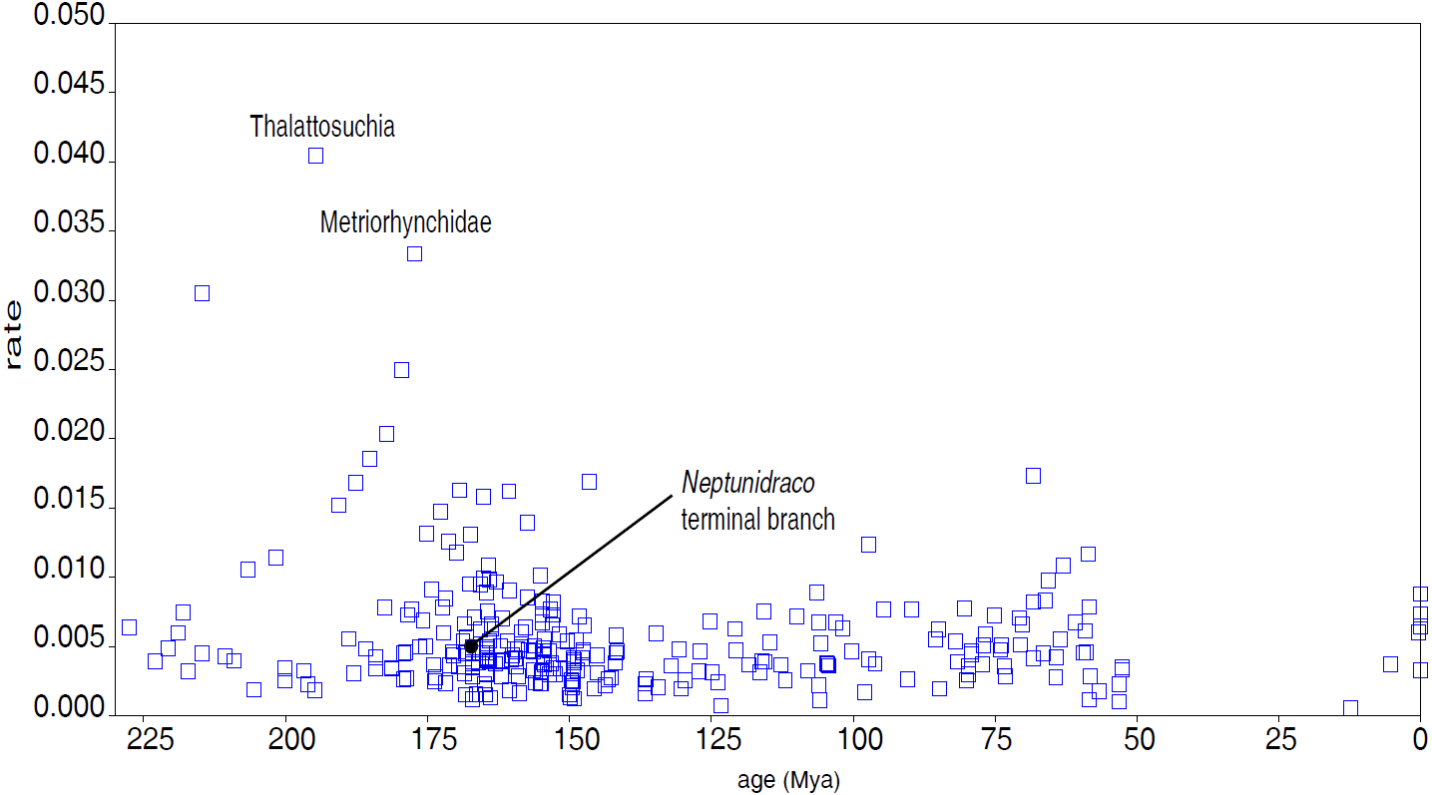
Divergence rate of the Crocodylomorph branches. Rate of divergence of the crocodylomorph branches (amount of change per branch per million year) reconstructed in the MCCT (Fig. 3) plotted over time (mean age of divergence of node).

The first Bayesian analysis inferred cladogenetic timing, estimated the frequency of potential anagenetic ancestors in the sample, and estimated the divergence rate (inferred amount of morphological divergence *per* branch *per* time unit) (Cau, 2017). The median ages of divergence from their immediate sister taxon for, respectively, Thalattosuchia, Metriorhynchidae and Metriorhynchinae are inferred at 201.75 Ma, 179.58 Ma and 177.34 Ma. The sister-group relationships inferred in the MCCT are in large majority reconstructed as cladogenetic patterns: although a direct ancestor-descendant relationships was inferred for a few sister-taxon couples recovered in the MCCT (e.g., between “Mr Leed’s Dakosaur” relative to the branch including the other members of Dakosaurina), the *pp* of these hypotheses is weak. The distribution of the median rate values in the MCCT indicates that the highest rate of evolutionary divergence was acquired at the root of Thalattosuchia (median rate inferred at 0.038 per My per branch) and at the root of Metriorhynchidae (median rate inferred at 0.030 per My year per branch). This result differs from the analysis of Cau & Fanti (2015), which found the highest rate of the thalattosuchian radiation for the *Neptunidraco* branch (which included MPG 6552). In the MCCT topology, the rate of divergence for the *Neptunidraco* branch (0.005 per My per branch) does not significantly differ from the background rate of the whole thalattosuchian clade (Fig. 5).

## Discussion

The use of alternative analytical methods (see Madzia & Cau, 2017) and the discrepancy in the relative phylogenetic interpretation of the RAVF crocodylomorphs which resulted highlight an intrinsic limit in the accuracy of any phyletic interpretation for these specimens, due to their highly fragmentary nature. Both the holotype of *N. ammoniticus* and MGP 6552 are confidently referred to Metriorhynchoidea, and the former shows unique features of Metriorhynchidae. Yet, the combination of features in MGP 6652 defines a grade (a paraphyletic group) of metriorhynchoids, and cannot be used to more accurately place this specimen in the clade. The less completely scored holotype of *N. ammoniticus*, on the contrary, cannot be more precisely referred relative to one of the two main metriorhynchid lineages because the phylogenetically-significant features visible in this specimen are homoplastic in Metriorhynchidae and are optimised as evolving independently at least twice among basal geosaurines and basal metriorhynchines. For example, the acute angle between the posterior processes of the frontal, present in *N. ammoniticus*, is optimised in all the topologies inferred by the parsimony analyses to evolve at least twice, being a synapomorphy of a clade of Geosaurinae including Geosaurini and of a clade of Metriorhynchinae including Rhacheosaurini (Young et al., 2010; Parrilla-Bel et al., 2013). All analyses using Implied Weighted parsimony produced the same topologies obtained using unweighted parsimony, which indicates that the unresolved positions of the RAVF specimens are not due to some bias in weighting settings. The phylogenetic placements of the RAVF specimens obtained by the Bayesian inference analyses partially overlap those obtained by the parsimony-based analyses. Several factors may produce discrepancies in the topologies recovered by parsimony and Bayesian inference methods performed with the same morphological data set (Lee & Palci, 2015; O’Reilly et al., 2016). The relative high incompleteness of the Italian specimens (all scored for <15% of the characters used in the data set) is expected to be less problematic for the Bayesian-based method relative to the parsimony analyses (Wiens, 2005; Wiens & Morrill, 2011; Wright & Hillis, 2014; O’Reilly et al., 2016; Puttick et al., 2017). Accordingly, the main result of the tip-dating Bayesian analysis, placing all RAVF specimens—including FMCR SP3839—in Metriorhynchoidea, is here followed. Note that the only character state supporting the referral of FMCR SP3839 to some neosuchian clade (i.e., number of dentary alveoli between 15 and 19; character statement 278.2) is also shared with basal metriorhynchoids, and thus cannot dismiss the referral to the latter lineage. Furthermore, the alternative placements of *Neptunidraco* relative to metriorhynchids and geosaurines cannot be solved based on the actual evidence. Even if the metriorhynchine placement results supported by a subset of the parsimony-based topologies and by both Bayesian-based analyses, the characters supporting this scenario are homoplasious among metriorhynchids and evolved convergently also in Geosaurinae, and cannot be used to dismiss the referral to the latter clade. Thus, the most conservative approach is to consider *N. ammoniticus* a Metriorhynchidae *incertae sedis*. Furthermore, with the exclusion of the subset of topologies placing FMCR SP3839 among neosuchians, discussed here, there is no evidence of a second clade of crocodylomorphs in the RAVF, because all known archosaurian material so far recovered from this formation since the 18th Century has consistently been referred to Thalattosuchia (Cau, 2014; Cau & Fanti, 2015). The most conservative interpretation of all available evidence is thus to refer all RAVF specimens to Metriorhynchoidea. The more unstable positions recovered for the two specimens previously referred to *Neptunidraco*, in one case (FMCR SP3839) likely biased by the extremely fragmentary nature of the material, prevent any robust assessment of their placement beyond the referral to Metriorhynchoidea.

One intriguing result of the Bayesian inference analysis performed here is that the rate of morphological divergence inferred for *Neptunidraco* is not significantly different from that of the majority of the thalattosuchian branches (*contra* Cau & Fanti, 2015; Fig. 5). Although such result is in part due to the updated character score of the Italian specimens (which may have removed spurious scores that inflated the amount of autapomorphies in the terminal branch used in the previous iterations; Cau & Fanti, 2015; Fanti et al., 2016), it is also likely a consequence of not forcing the RAVF specimens into a single terminal unit *a priori* (which might have resulted in the inclusion in the analysis of a possible chimera with an artificial score combination)*.* In the updated analysis, the peaks in morphological divergence rate during thalattosuchian evolution are inferred along the basal divergence of the clade from other crocodylomorphs, and at the root of the metriorhynchid radiation (Figs. 3 and 5): this scenario suggests that the full adaptation to an aquatic lifestyle occurred through two distinct and relatively rapid ecomorphological transitions, the first occurring at the very beginning of the Jurassic, between 201 and 195 Ma (leading to the last common ancestor of teleosauroids and metriorhynchoids), the other between 190 and 177 Ma (leading to the last common ancestor of metriorhynchines and geosaurines) (see Young et al., 2010). A possible bias due to a major focus on synapomorphies of Thalattosuchia compared to those of other clades included in the data set of Ösi et al. (2018) cannot be ruled out for explaining the unusually-high divergence rates inferred along the earliest branches of Thalattosuchia, compared to the rest of Crocodylomorpha. Nevertheless, it is unlikely that the results were significantly affected by such character sample artifact, because the rate of divergence inferred among the non-thalattosuchian branches was not significantly lower than the background rate of the entire crocodylomorph group (Fig. 5). Furthermore, if the taxon sample significantly affects the rate of divergence among thalattosuchians, it is expected that removing most of the non-thalattosuchian taxa, the re-calculated rates in Thalattosuchia will be artificially inflated. An alternative version of the Bayesian inference analysis performed here, which included a dramatically reduced taxon sample among non-thalattosuchian taxa (i.e., only six non-thalattosuchians were included in the data matrix), inferred approximately the same pattern for the tempo and mode of thalattosuchian basal radiation: even in that test, the highest rates of divergence were inferred along the basal branches of Thalattosuchia (with values comparable to those obtained in the first analysis), demonstrating that the values inferred among the latter clade are not biased by taxon sample artifacts, but instead reflect the peculiar natural history of Thalattosuchia compared to the other main crocodylomorph lineages (i.e, its unique *bauplan* and its rapid and successful diversification during the Jurassic). These results indicate that during the first part of the Jurassic, thalattosuchians markedly diverged from the ancestral crocodylomorph condition (“sphenosuchian-protosuchian grade”), earlier and more radically than the other main lineages (i.e., notosuchians and neosuchians), exploring and adapting to a completely novel ecomorphological regime (Young et al., 2010).

## Conclusions

A revision of the affinities of the Rosso Ammonitico Veronese Formation thalattosuchians is here provided. The specimens are critically re-scored and included simultaneously in a phylogenetic data set for the first time. The specimen-level analyses do not confirm the unambiguous referral of *Neptunidraco* to Geosaurinae found in previous analyses of Thalattosuchia. With its unique mix of derived and plesiomorphic features and its Bathonian age pre-dating all other known metriorhynchids, *Neptunidraco* is a key taxon in our reconstruction of the tempo and mode of metriorhynchoid macroevolution. Given the fragmentary nature of the holotype specimen of *N. ammoniticus*, a more accurate referral of this taxon than to Metriorhynchidae is not possible based on the currently known material. The diagnosis of *N. ammoniticus* is here emended. The results of the analyses support alternative placements for the other specimens from the RAVF: although their referral to *Neptunidraco* cannot be unambiguously dismissed by the parsimony analysis, they are conservatively considered as Metriorhynchoidea *incertae sedis*. The unusually high rate of divergence inferred for the *Neptunidraco* branch in the previous iterations of the Bayesian inference analysis of Thalattosuchia was likely biased by the *a priori* inclusion of MGP 6552 in *Neptunidraco*: this result highlights the importance of taxon sampling and explicit taxon unit definitions in morphological phylogenetics, and reinforces the role and utility of specimen-level analysis for the reconstruction of the evolutionary patterns in paleontology.

##  Supplemental Information

10.7717/peerj.7364/supp-1 Supplemental Information 1 Phylogenetic data matrix in .tnt format Data matrix used for the parsimony analyses.

10.7717/peerj.7364/supp-2 Supplemental Information 2 Bayesian inference data set

10.7717/peerj.7364/supp-3 Figure S1 MCCT of Crocodylomorpha Maximum Clade Credibility Tree reconstructed by the Bayesian inference analysis. Branches colored according to median rate of morphological divergence inferred (note highest values at the root of the thalattosuchian and metriorhychid radiations). Tip ages based on median value inferred: actually recorded extent of terminal taxa not reported. Note that additional Early Cretaceous material (not included in the analysis) may extend the metriorhynchid record to the Aptian-Albian (see Chiarenza et al., 2015), and that the timing of extant clade diversification is delayed by absence in the sample of fossil members of the crown-group. The numbers above the branches indicate the posterior probability values ¡0.5. The RAVF specimens are indicated by the grey rectangles.

10.7717/peerj.7364/supp-4 Figure S2 Half-compact tree of the post-burnin trees sampled by the Bayesian inference analysis not integrating the stratigraphic information RAVF specimens indicated by grey rectangles.

## Additional Information and Declarations

### Data Availability

The following information was supplied regarding data availability: The the phylogenetic data matrices are provided in the Supplemental Files.
