# Supplementary figures and images for: A revision of the diagnosis and affinities of the metriorhynchoids (Crocodylomorpha, Thalattosuchia) from the Rosso Ammonitico Veronese Formation (Jurassic of Italy) using specimen-level analyses
Source: PeerJ. 2019 Jul 22;7:e7364. doi: 10.7717/peerj.7364 (PMC6712679; doi:10.7717/peerj.7364)

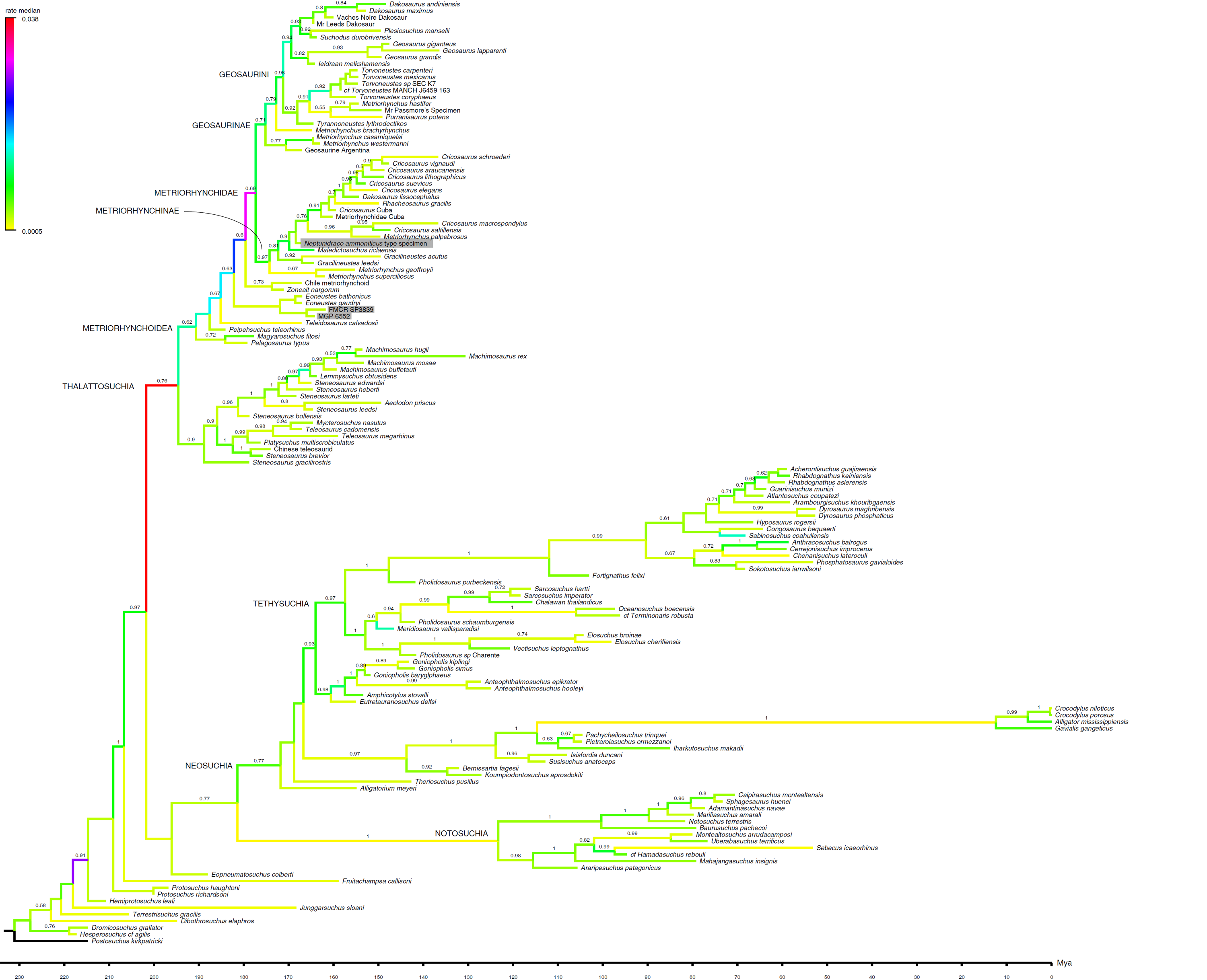

Supplement: Figure S1 — Maximum Clade Credibility Tree reconstructed by the Bayesian inference analysis. Branches colored according to median rate of morphological divergence inferred (note highest values at the root of the thalattosuchian and metriorhychid radiations). Tip ages based on median value inferred: actually recorded extent of terminal taxa not reported. Note that additional Early Cretaceous material (not included in the analysis) may extend the metriorhynchid record to the Aptian-Albian (see Chiarenza et al., 2015), and that the timing of extant clade diversification is delayed by absence in the sample of fossil members of the crown-group. The numbers above the branches indicate the posterior probability values ¡0.5. The RAVF specimens are indicated by the grey rectangles. [file peerj-07-7364-s003.png]

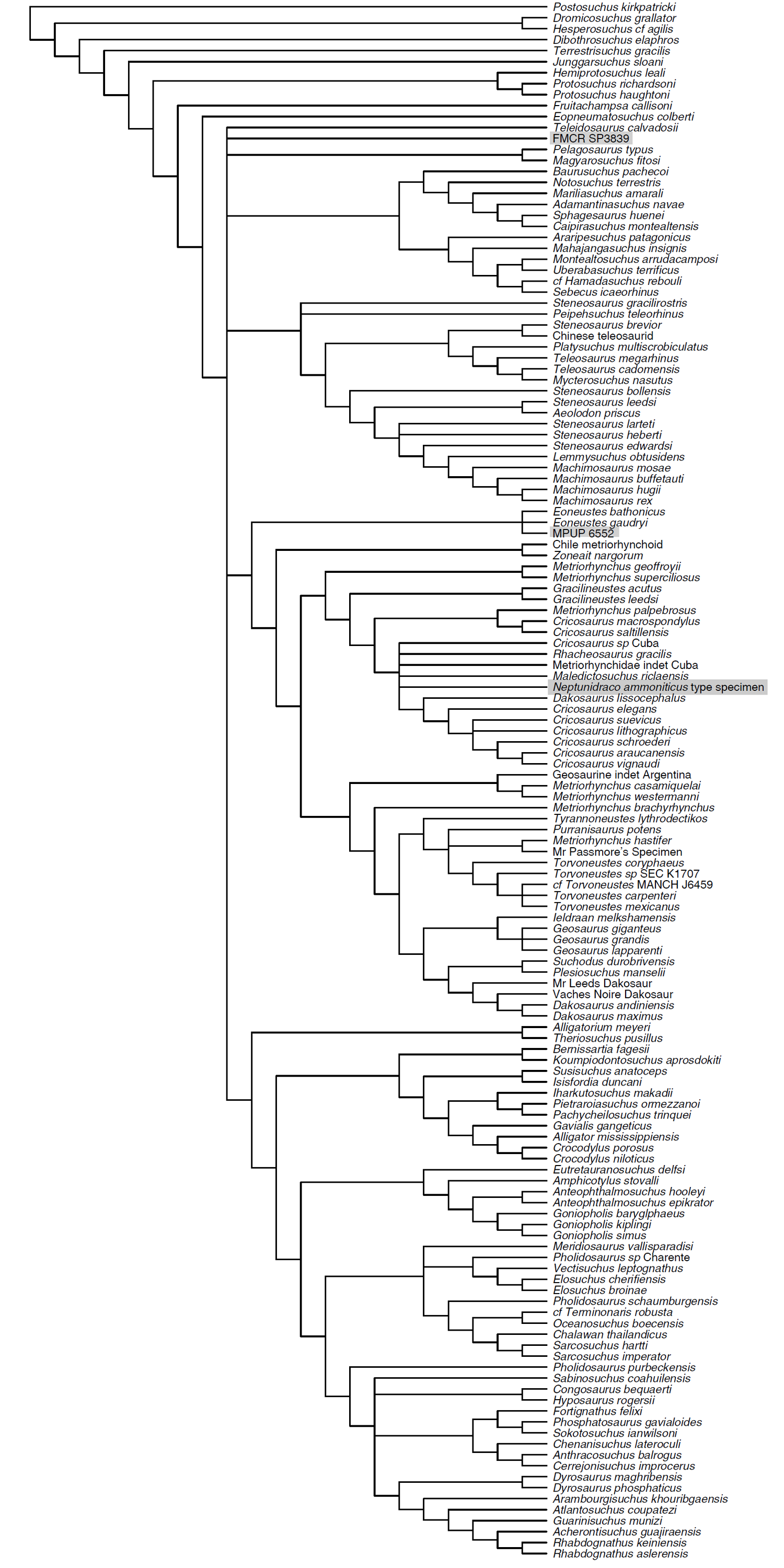

Supplement: Figure S2 — RAVF specimens indicated by grey rectangles. [file peerj-07-7364-s004.png]
